# Growth and Morphogenesis during Early Heart Development in Amniotes

**DOI:** 10.3390/jcdd4040020

**Published:** 2017-11-22

**Authors:** Kenzo Ivanovitch, Isaac Esteban, Miguel Torres

**Affiliations:** 1 Developmental Biology Program, Centro Nacional de Investigaciones Cardiovasculares (CNIC), 28029 Madrid, Spain; isaac.esteban@cnic.es; 2 Departamento de Ingeniería Electrónica, ETSI de Telecomunicaciones, Universidad Politécnica de Madrid, 28040 Madrid, Spain

**Keywords:** heart tube, cardiac crescent, cardiac differentiation, first heart field, second heart field, cardiomyocyte, live-imaging

## Abstract

In this review, we will focus on the growth and morphogenesis of the developing heart, an aspect of cardiovascular development to which Antoon Moorman and colleagues have extensively contributed. Over the last decades, genetic studies and characterization of regionally regulated gene programs have provided abundant novel insights into heart development essential to understand the basis of congenital heart disease. Heart morphogenesis, however, is inherently a complex and dynamic three-dimensional process and we are far from understanding its cellular basis. Here, we discuss recent advances in studying heart morphogenesis and regionalization under the light of the pioneering work of Moorman and colleagues, which allowed the reinterpretation of regional gene expression patterns under a new morphogenetic framework. Two aspects of early heart formation will be discussed in particular: (1) the initial formation of the heart tube and (2) the formation of the cardiac chambers by the ballooning process. Finally, we emphasize that in addition to analyses based on fixed samples, new approaches including clonal analysis, single-cell sequencing, live-imaging and quantitative analysis of the data generated will likely lead to novel insights in understanding early heart tube regionalization and morphogenesis in the near future.

## 1. Early Cardiac Development

**Figure 1:**
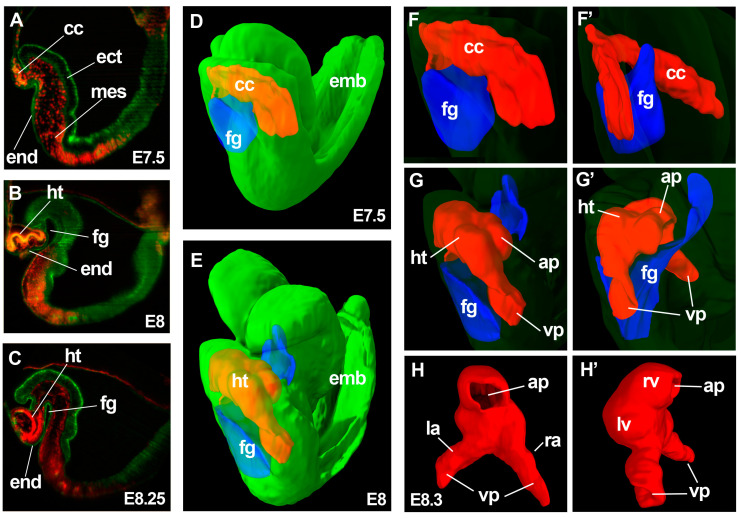
3-dimensional analysis of heart tube formation. (**A**–**C**) show optical sections of 3D reconstructions obtained by light-sheet microscopy from E7.5 (**A**); E8 (**B**) and E8.25 (**C**) embryos. *Rosa26GFP*; *Mesp1^cre/+^*; *R26Rtdtomato* embryos are shown in (**A**,**B**); while a *Mesp1^cre/+^*; *R26RmT/mG* embryo is shown in (**C**); Ectoderm and endoderm are shown in green and mesoderm in yellow (**A**,**B**) or red (**C**); (**D**,**E**) show simultaneous display of the 3D reconstructions of the foregut pocket (blue), cardiac crescent (red) and whole embryo (green) from E7.5 (**D**) and E8 (**E**) embryos; (**F**–**G’**) show magnified 3D views of the cardiac region of the embryo 3D models in (**D**,**E**); The whole-embryo volume (green) has been transparented for appreciation of the details of the cardiac region. (**F**,**G**) show ventro-lateral views and (**F’**,**G’**) show dorso-lateral views; (**H**,**H’**) a reconstruction of the linear heart tube from an E8.3 embryo; (**H**) shows a dorsal view and (**H’**) a lateral view. The image indicates the structures derived at this stage from the FHF: left ventricle and part of the atria and SHF: right ventricle and part of the atria. **ht**, heart tube; **cc**, cardiac crescent; **fg**, foregut pocket; **end**, endoderm; **mes**, mesoderm; **ect**, ectoderm; **emb**, embryo; **ap**, arterial pole; **vp**, venous poles; **lv**, left ventricle primordium; **rv**, right ventricle primordium; **la**, left atrium primordium; **ra**, right atrium primordium.

Cardiac precursors are found shortly after gastrulation within the mesodermal component of the splanchnopleural layer of the anterior-most lateral plate [1,2,3]. This area is called the cardiogenic area and is formed by early gastrulating embryonic mesoderm. The cardiogenic area is single and crescent-shaped in the mouse and bilaterally paired in human and avian embryos [1]. In the mouse, the cardiac mesoderm first colonizes the rim between the head folds and the extraembryonic region, at this stage lying at the most anteriolateral embryonic region, forming a horseshoe-shaped primordium. In the human and avian embryo, in contrast, two cardiac primordia are formed bilaterally without continuity across the anterior midline. The outermost rim of the cardiac mesoderm closer to the extra-embryonic region is the first area to show signs of differentiation towards the cardiomyocyte fate and is known as first heart field (FHF), which, in the mouse is arranged in a crescent shape and thus named cardiac crescent [3] (Figure 1). 

Subsequently, and as part of the general embryonic folding process that brings the endoderm to the inside of the embryo, cardiac precursors are placed at their definitive position posterior and ventral to the head. During these movements, the heart forming regions are always in close contact with the pharyngeal endoderm, being placed ventrally to the foregut pocket (Figure 1). The rest of mesodermal cardiac precursors positioned posteromedially and immediately adjacent to the cardiac crescent in the splanchnopleura are known as the second heart field (SHF) and remain undifferentiated at this stage [4,5,6,7]. The FHF gives rise to posterior structures of the primitive heart tube, including the left ventricle and most of the atria. The recruitment of FHF precursors to the heart tube takes place “all-at-once” by simultaneous folding and remodeling of the splanchnopleural mesoderm, but the SHF remains in contact with the endoderm and is maintained as a pool of undifferentiated proliferating cardiac precursors for about two days in the mouse. During this period, the SHF progressively contributes new cardiac precursors that form the right ventricle and outflow tract (OFT) at the arterial pole, and part of the atria and inflow tract at the venous pole [3,8,9,10]. The equilibrium between the proliferative/undifferentiated status of SHF precursors and their differentiation is essential to sustain proper heart formation. The negative regulatory feedback loop between Nkx2.5 and BMP [11] and the cooperation of the transcription factor Hopx with BMP to block Wnt signaling [12] play essential roles in maintaining this equilibrium.

A set of transcription factors essential for cardiac specification is expressed in cells becoming allocated to the cardiac mesoderm. Some of these, like Gata-4, Nkx2.5, Mef2c and Islet1, are expressed by most cardiac precursors in the FHF and SHF [13,14,15], while others are restricted to regions contributing to specific parts of the heart; Tbx5 is preferentially expressed in the FHF [16]; Hand2 in all anterior SHF derivatives, including the right ventricle and outflow tract [17]; Tbx1 in the anterior SHF and Tbx18 in the posterior-most SHF subpopulation [18,19,20]. 

In light of these findings, heart congenital disease is now better understood in terms of its developmental origins [21]. Several cardiac congenital defects have been linked to mutations in genes encoding cardiac developmental transcription factors [22] and several of them, like Tbx1, involved in DiGeorge syndrome [23], are related to factors relevant in SHF development.

In human and avian embryos, the initial cardiac fields occupy a paired bilateral position and do not span across the midline anterior to the head-forming region. Formation of the primary heart tube in these species thus involves the fusion of two primordial tubes initially formed bilaterally [24,25]. A particularly relevant contribution of Moorman and colleagues to the compared understanding of cardiac development in amniotes is the generation of a 3D interactive atlas of human development, including a detailed study of the cardiovascular system [26]. In the mouse, as mentioned above, the first cardiac cell differentiation events take place in a cardiac crescent already continuous across the midline and therefore does not require the same morphogenetic movements that take place in the human and avian embryos [1]. Cardiac progenitors are, however, also located bilaterally prior to this early differentiation phase, since they migrate bilaterally from the primitive streak and merge at the anterior midline. Each bilateral heart forming region is capable of forming a differentiated chambered heart on its own [14,27,28,29] when midline fusion fails. The heart is therefore a bilateral structure as well in the mouse. By the first signs of contractility (around E8 in the mouse), the cardiac tube is just a fold of the splanchnic mesodermal layer that is not closed dorsally [30]. Thus, although commonly called “heart tube”, at this stage only the endocardium forms a tube, while the prospective myocardium is a hemitube with its open side sitting on the endoderm. Endocardial cells beneath this fold, however, soon form a sealed tube enclosed between the endoderm and the primary cardiac tube, capable of sustaining fluid circulation as soon as contractions appear. With the closure of its dorsal aspect, the primitive tube derived from the FHF is finished around 18 days in the human embryo (E8.5 in the mouse) [24].

## 2. Antero-Posterior Patterning of the Primitive Heart Tube

The heart at E8 in the mouse, (corresponding to Carnegie stage 9 in human and Hamburger-Hamilton stage 9 (HH9) in chicken), is composed, at the cranial part of the tube by the primordium of the left ventricle, and caudally by two arms representing the precursors of the atrial chambers [31]. Precursors of the right ventricle will be added subsequently at the most cranial part of the HT by differentiation of SHF cells [32] (E8.5 in the mouse). Thus, the primary HT is patterned along the cranio-caudal axis and this is important for the acquisition of the atrial and ventricular fate later in development. This was recognized by early work from Moorman and colleagues, who studied the three-dimensional distribution of atrial and ventricular myosin isoforms in the developing heart tube of the chick [33]. Patterning is first detectable by the differential expression of transcription factors. For example, *Irx4* is expressed more cranially, in the regions forming the ventricles and its misexpression activates the ventricular phenotype along the antero-posterior heart tube axis [34]. The T-box transcription factor Tbx5 instead has graded expression levels along the cranio-caudal axis of the HT, with expression being more intense caudally, in the left ventricle and atrial primordia. Accordingly, it is required for the expression of markers expressed preferentially in these posterior chambers primordial, such as the atrial natriuretic peptide–coding gene *Nppa* and Connexin40 (*Gja5*) [35,36]. Understanding the function of Tbx5 in HT AP patterning is especially important since haploinsufficiency of this gene has been linked to the Holt-Oram syndrome, a complex congenital condition causing [35,37,38] hypoplastic left heart, among other manifestations.

Classical transplantation studies in both mouse and chick also gave rise to important insights as they show that presumptive atrial (posterior) cells can acquire ventricular (anterior) phenotype properties when placed in the prospective ventricular domain [39]. Moreover, the ability of the cells to switch from a ventricular to an atrial identity seems to be retained up to stage E12.5 in the mouse [40], long after the initial differentiation of the cardiomyocytes takes place. These experiments show that the diversified phenotype of cardiomyocytes is not fixed and can be influenced by positional effects. In other words, the cellular/signaling environment may be more important than autonomous mechanisms in specifying the identity of the cells [41]. Retinoic acid (RA) signaling from the lateral plate mesoderm has been shown to define the posterior identity of the heart tube [42,43,44]. In addition, the anterior intestinal portal endoderm, in addition to promoting the cardiac fate, can also pattern the heart tube by specifying ventricular and suppressing atrial identity [45]. All these observations are consistent with a model where a common population of cardiomyocytes may be patterned along the AP axis during the formation of the initial heart tube by extrinsic signals. Cardiac lineage specification however may be an earlier event. Fate map analyses in chicken demonstrated that distinct types of cardiac mesoderm arise in an orderly fashion along the AP axis of the primitive streak, with cardiac ventricle progenitors deriving from anterior cardiac precursors while atria progenitors arising from more posterior regions [46,47,48,49,50]. The specification of cardiac precursors according to their position in the primitive streak could be explained exclusively by the extrinsic influences they experience during migration and at their definitive position in the cardiac forming region [47,51,52]. Nonetheless, in favor of a role for intrinsic programs in this process, a population of cardiac progenitors that transiently express *Foxa2* during mouse gastrulation has been shown to mostly contribute to ventricles [53].

As development progresses, between 3.5 and 7 weeks of human development (E8.5 to E11.5 in the mouse and HH9 to 18 in chicken), the heart undergoes extensive growth and morphological modifications, leading to the formation of a partially septated four-chambered heart equipped with a set of immature valves. Subsequent OFT subdivision and complete inter-ventricular and atrial septation lead, around the 12th week in humans (~E16.5 in the mouse; HH34 in chicken), to a heart bearing the gross morphological organization of a definitive adult heart [24]. During this process the cardiac neural crest cells invade the OFT and play critical roles in its septation and morphogenesis [54]; see [1] for an overview.

## 3. Chamber Formation

**Figure 2:**
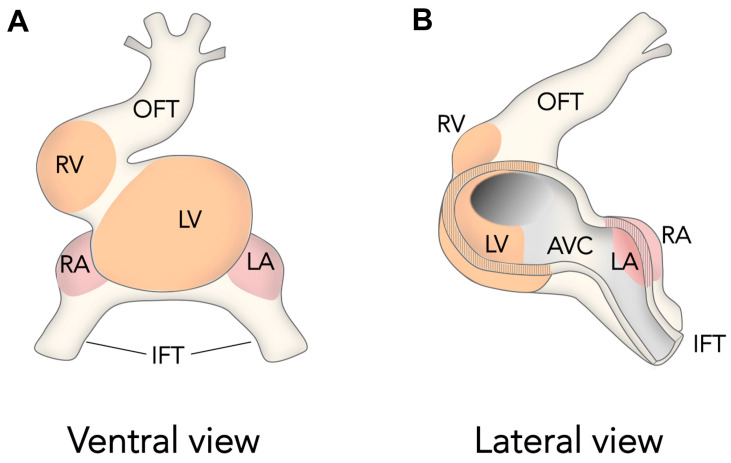
The ballooning Model for Cardiac Chamber Initiation. Schemes show ventral (**A**) and lateral–partially open–(**B**) views of the looping amniote heart. The schemes are inspired in previous Figures published by Anton Moorman and colleagues [55]. Note how chambers develop at localized areas of the outer curvature of the heart tube and not form cylindrical segments of the heart tube. **OFT**, outflow tract, **RV**, right ventricle, **LV**, left ventricle, **RA**, right atrium, **LA**, left atrium, **IFT**, inflow tract, **AVC**, atrio-ventricular canal.

The initial heart tube is composed of primary myocardium, which shows poor contractility and low conduction velocity. The inside of the primitive cardiac tube is lined by the endocardium, which is separated from the myocardium by a mass of cardiac jelly. While the classical view of heart development considered a segmental model for the contribution of the early heart tube to chamber formation, essential insight from the pioneering work of Moorman and colleagues changed this view, providing a new and widely accepted model for this process; the ballooning model [55] (Figure 2). This model has received support from further detailed studies of the 3D expression patterns of chamber and non-chamber cardiac markers, which indicated that the activation of chamber primordia is not segmental but localized to discrete regions of the outer curvature of the looping heart [56] (Figure 2). 

The study of chamber-specific markers by Moorman and colleagues has also been essential for understanding how the molecular programs driving cardiomyocyte diversification are established. The delimitation of the chamber-forming regions within the cardiac tube is governed by a transcriptional network with a predominant role for the T-box family factors Tbx2, 3, and 20 [2,18,57,58,59,60]. The first signs of chamber initiation are the increase in local proliferation and the activation of the atrial natriuretic peptide–coding gene *Nppa* [61]. Tbx20 is expressed throughout the heart tube and is essential for chamber formation. In contrast, Tbx2 and Tbx3 are both expressed specifically in the non-chamber myocardium and are needed to maintain the primitive myocardial character and repress chamber formation in these regions. Tbx2/3 are transcriptional repressors themselves and may also recruit HDACs to promote repressed chromatin states in chamber-promoting genes. Sustained high levels of BMP signaling are responsible for the maintenance of Tbx2/3 expression in the non-chamber myocardium, whereas Tbx20 repression of BMP signaling excludes Tbx2/3 from the chamber-forming regions, which allows the development of the working myocardium in these areas.

On the other side, pioneering 3D reconstructions of the developing chick, human and mouse developing hearts at cellular resolution allowed Moorman and colleagues to investigate for the first time the evolution of the 3D patterns of cardiac cell proliferation [31,62,63]. These studies represent the first serious attempt to a quantitative description of cardiac development in amniotes and included measurements of cell numbers and proliferative activity at a spatial and temporal resolution never achieved before. Importantly, the studies were performed in three different amniote species, including human embryos, which provides a solid and comparative basis for understanding heart development. These studies indicated that chamber formation can be detected by local activation of proliferation in the heart tube. In the human and chicken embryo, this proliferation is reactivated in cardiomyocytes after a phase of proliferation arrest during FHF differentiation [62,63], while in the mouse embryo proliferation seems not to be completely interrupted at any stage [31]. Chamber formation thus takes place by hyperproliferation of discrete regions leading to local ballooning of the linear tube walls. In addition, these quantitative studies at cellular resolution also indicated a role for cell size and shape changes during chamber formation [64]. Interestingly, proliferation reactivation seems to take place at a single point of the early heart tube and to expand concentrically from this point [62], however the mechanisms that specify the spatial regulation of this reactivation remain unknown. The ballooning model for heart chamber formation has profound implications to the understanding of how the mature pattern of cardiac circulation is established as, in contrast to the segmental model, it establishes that both ventricles are connected to atria from their first appearance [65]. The ballooning model, however, does not extend into how the final growth and thickening of the ventricular wall produces the mature myocardium at the final stages of embryonic development. In zebrafish, from the juvenile to adult stage, the ventricular wall of the heart thickens dramatically by proliferation of a small number of cells (around 8/heart) emerging from the trabeculae through an “inside-out” mechanism [66]. Although no evidence exists for a similar mechanism in amniotes, in the future it would be interesting to address the possible evolutionary conservation of this aspect of ventricle formation.

The first discernable chamber is the left ventricle, initially located in a central posterior position of the linear tube, between the outflow and inflow tracts [31,62,63]. This stage is transitory as, immediately after, heart looping takes place. During heart looping, the cardiac tube undergoes a dextral bending that positions the right ventricle primordium in its definitive position with respect to left ventricle. Heart looping is concomitant with continuous chamber growth and progressive addition of the right ventricle primordium to the arterial pole and the atria primordia at the venous pole [31,55]. The forming chamber myocardium progressively acquires fast conduction and high contractility and increasingly differentiated sarcomeric structures [65]. In contrast, the non-chamber myocardium retains immature features and, after extensive repositioning of the chambers, it contributes to form the base of the ventricles and the atrioventricular (AV) valves as well as the outflow and inflow tracts [65]. In addition, cardiac jelly is excluded from the forming chambers but remains at the outflow and AV canal areas, where it exerts a resistance to blood flow that prevents blood reflux until proper valves are formed [67].

## 4. Towards New Quantitative and Dynamic Approaches to Understanding Heart Development

The pioneering work of Moorman and colleagues has highlighted the necessity of detailed quantitative 3D analyses at cellular resolution for a deep understanding of cardiac development. The extraordinary dynamism of early heart formation demands detailed 3D approaches at cellular level, including cell proliferation, migration, rearrangements, polarity, differentiation and shape/size changes. Gene expression patterns need also to be interpreted in their correct 3D context, as illustrated by the discovery of the ballooning model. While Moorman and colleagues worked by reconstructing 3D images from serial histological sections, it is likely that new optical approaches such as light-sheet microscopy [68,69,70] or episcopic imaging [71] combined with optical clearing of fixed embryos [72] will provide further abilities in 3D analysis in the near future. Light-sheet microscopy utilizes a thin sheet of laser light that provides optical sectioning of the sample and fluorescent light collection perpendicular to the illumination plane. This method is particularly promising since this allows to image thick samples in 3D in a fast manner and at high resolution by reducing out-of-focus signal. The speed and high penetration properties of this technique has provided important advantages for live analysis of heart development in zebrafish [73]. An example of 3D reconstruction of whole embryos and cardiac forming regions using light-sheet microscopy is provided in Figure 1. On the other side, episcopic microscopy, including episcopic fluorescence image capturing (EFIC) and high-resolution episcopic microscopy (HREM) [74], is based on serially imaging the surface of histology blocks as sections are being sliced out. While this approach is only possible for fixed samples, it renders large 3D volumes at unprecedented voxel resolution and high fidelity at histological levels [74].

An important experimental approach in understanding the cellular basis of cardiac development is clonal analysis [75,76], which allows not only the determination of lineage relationships but also the timing of cardiac precursor incorporation to the heart tube and the local variations in growth anisotropy and proliferation [77,78,79,80,81]. Pioneering studies in the chick using viral vectors for clonal analysis provided essential data on how cardiomyocyte lineages organize during heart chamber formation [82,83,84,85]. More recently, genetic tracing has been used for clonal analyses in the mouse and shed light on the organization of cardiac precursors in first and second heart fields [80]. The combination of these approaches with single-cell sequencing has allowed to refine our understanding of FHF versus SHF precursor specification in the early cardiac mesoderm [86,87]. 

Interestingly, not only the clonal analyses but also the anisotropy of clonal growth in the early heart tube, and the observation that planar polarity mutants provoke morphological defects in early heart tube suggests that the local coordination of cardiomyocyte polarity and proliferation patterns is a fundamental player in cardiac morphogenesis. Given the highly dynamic and complex 3D arrangement of heart tube formation, the quantitative analysis of cardiomyocyte local organization and division patterns represents a very important challenge. An important contribution to the understanding of this complex process came from a detailed quantitative analysis of polarity in 3D in the embryonic mouse heart, which revealed local patterns of cell coordination during chamber formation [88]. In this work, the authors developed new computerized approaches for the study of spatial coordination of cellular behavior. This allowed to show that the centrosome-nucleus axes and the cell division axes are biased in a plane parallel to the outer surface of the heart, with a minor transmural component. These axes were shown to align locally with respect to a plane tangential to the heart surface. This study highlights the relevance of developing new observation methods and new quantitative analytical tools in order to understand the cellular basis of cardiac development.

Given the dynamic nature of heart development, a further natural step in the progress on new approaches to understanding heart development is time-lapse analysis of heart formation in living embryos. Live analysis allows not only capturing tissue dynamics but, with the use of appropriate fluorescent reporters and advanced 3D microscopy, the tracing of individual cells and the description of their movements, lineages, differentiation patterns, shape and size changes and proliferative activity. Using 3D reconstruction of fixed samples (Figure 1) it is possible to recognize morphological structures across several stages of heart tube formation and infer the morphogenetic remodeling events. However, the cellular composition of these morphological structures may not be constant, as cells may change their relative positions and dynamically reorganize, crossing the boundaries between the observable morphological structures and eventually adapting their expression programs to the changing environment. Live-analysis will therefore hold special value in addressing how the dynamics of cell fate acquisition relates to the morphogenesis of the heart. While mammalian development naturally takes place in utero, embryo culture methods allow live analysis of the post-implantation mouse embryo by confocal [89,90,91,92] and light-sheet microscopy [93,94,95] in a reliable manner. The effective window for live analysis of the postimplantation mouse embryo spans from a couple of days before gastrulation (E4.5) until about E8.75, when embryo turning and dependence on placental circulation start to represent a limitation to in vitro development. This time window includes early cardiac development from the specification of cardiac precursors at gastrulation through their differentiation and incorporation to the primitive heart tube up to the heart looping stage. The lordotic disposition of the early mouse embryo exposes the cardiac crescent and early heart tube to the observer at the anterior region of the early embryo (Figure 1), which facilitates microscopic approaches for recording heart development. The wealth of transgenic fluorescent reporters available in the mouse model also provides an important advantage for live analysis approaches in this model. 

Taking advantage of these characteristics, a recent study reports the first live analysis of the initiation of heart tube electrical activity [30]. In this study Tyser and colleagues used calcium reporters to describe the first appearance of calcium sparks in cardiomyocytes at the cardiac crescent and how these evolve to waves as the cardiac tissue matures. Initially, randomly distributed spontaneous asynchronous Ca^2+^ oscillations were observed in the cardiac crescent before overt beating is detected. Nascent contraction was detected at around E8.0 and was associated with sarcomeric assembly and rapid Ca^2+^ transients. Interestingly, the Na^+^–Ca^2+^ exchanger NCX1 was required for this activity and its blockade prevented progression in the activation of the cardiomyocyte differentiation program. These results highlight the relevance of live analysis, and not only describe the physiology of early cardiac differentiation, but link physiology to differentiation from the very first steps of heart formation.

In a further application of live analysis to cardiac development, we have recently established multiphoton confocal analysis for the study of heart tube formation at cellular resolution [96]. The approach allowed us to globally track FHF and SHF cell populations by using fluorescent reporters. Furthermore, the combination of live reporters of FHF differentiation with the use of frequency-controlled randomly activated fluorescent reporters allowed the tracking of single cells and the determination of their trajectories, differentiation and proliferative patterns [96]. This study has provided new insights into the temporal regulation of FHF and SHF precursor specification and differentiation, revealing alternating phases of differentiation and morphogenesis. While FHF and SHF are adjacent cell populations, during the morphogenetic events that convert the cardiac crescent in the primitive heart tube, SHF precursors are blocked from differentiating. SHF cells only resume differentiation when linear heart tube morphogenesis is about complete and they do so by incorporating at both poles and to the dorsal seam of the heart tube. This live analysis reveals tissue-level coordination between morphogenesis and differentiation during HT formation and provides a new framework to understand early heart development.

We foresee that in the near future quantitative approaches at cellular resolution, as envisioned and pioneered by Prof Antoon Moorman, will represent a fundamental trend for advancing the understanding of cardiac development. The new developments will exploit new 3D and 3D + t approaches in the mouse and other species but will also essentially require the incorporation of new microscopy, analytic and computer modeling methods, calling for the involvement of multidisciplinary teams able to implement these complex approaches.
