# Correction: Ivanovitch et al. Growth and Morphogenesis during Early Heart Development in Amniotes. *J. Cardiovasc. Dev. Dis.* 2017, *4*, 20

**DOI:** 10.3390/jcdd9100360

**Published:** 2022-10-20

**Authors:** Kenzo Ivanovitch, Isaac Esteban, Miguel Torres

**Affiliations:** 1 Developmental Biology Program, Centro Nacional de Investigaciones Cardiovasculares (CNIC), 28029 Madrid, Spain; 2 Departamento de Ingeniería Electrónica, ETSI de Telecomunicaciones, Universidad Politécnica de Madrid, 28040 Madrid, Spain

## Additional Affiliation

In the published publication [1], there was an error regarding the affiliation for Isaac Esteban. In addition to affiliation 1, the updated affiliation 2 should include the following: Departamento de Ingeniería Electrónica, ETSI de Telecomunicaciones, Universidad Politécnica de Madrid, 28040 Madrid, Spain. The authors state that the scientific conclusions are unaffected. This correction was approved by the Academic Editor. The original publication has also been updated.

## References

[1] Ivanovitch K., Esteban I., Torres M. (2017). Growth and Morphogenesis during Early Heart Development in Amniotes. J. Cardiovasc. Dev. Dis..

